# Sub-Chronic Consumption of Dark Chocolate Enhances Cognitive Function and Releases Nerve Growth Factors: A Parallel-Group Randomized Trial

**DOI:** 10.3390/nu11112800

**Published:** 2019-11-16

**Authors:** Eri Sumiyoshi, Kentaro Matsuzaki, Naotoshi Sugimoto, Yoko Tanabe, Toshiko Hara, Masanori Katakura, Mayumi Miyamoto, Seiji Mishima, Osamu Shido

**Affiliations:** 1 Department of Environmental Physiology, Faculty of Medicine, Shimane University, Izumo 693-8501, Japan; tanabey@med.shimane-u.ac.jp (Y.T.); physiol1@med.shimane-u.ac.jp (T.H.); o-shido@med.shimane-u.ac.jp (O.S.); 2 Department of Physiology, Graduate School of Medical Science, Kanazawa University, Kanazawa 920-8640, Japan; 3 Department of Nutritional Physiology, Faculty of Pharmaceutical Sciences, Josai University, Sakado 350-0295, Japan; mkatakur@josai.ac.jp; 4 School of Nursing, Faculty of Medicine, Shimane University, Izumo 693-8501, Japan; mmiyamot@med.shimane-u.ac.jp; 5 Central Clinical Laboratory, Shimane University Hospital, Izumo 693-8501, Japan; mishima@med.shimane-u.ac.jp

**Keywords:** dark chocolate, theobromine, nerve growth factor, cognitive function, subchronic effect, young-adult, Stroop color word test, digital cancellation test

## Abstract

Previous research has shown that habitual chocolate intake is related to cognitive performance and that frequent chocolate consumption is significantly associated with improved memory. However, little is known about the effects of the subchronic consumption of dark chocolate (DC) on cognitive function and neurotrophins. Eighteen healthy young subjects (both sexes; 20–31 years old) were randomly divided into two groups: a DC intake group (*n* = 10) and a cacao-free white chocolate (WC) intake group (*n* = 8). The subjects then consumed chocolate daily for 30 days. Blood samples were taken to measure plasma levels of theobromine (a methylxanthine most often present in DC), nerve growth factor (NGF), and brain-derived neurotrophic factor, and to analyze hemodynamic parameters. Cognitive function was assessed using a modified Stroop color word test and digital cancellation test. Prefrontal cerebral blood flow was measured during the tests. DC consumption increased the NGF and theobromine levels in plasma, enhancing cognitive function performance in both tests. Interestingly, the DC-mediated enhancement of cognitive function was observed three weeks after the end of chocolate intake. WC consumption did not affect NGF and theobromine levels or cognitive performance. These results suggest that DC consumption has beneficial effects on human health by enhancing cognitive function.

## 1. Introduction

During the last decade, several studies have demonstrated that cacao-containing foods such as chocolate and cocoa may have beneficial effects on human health [1,2,3,4]. Although cacao is rich in fat and carbohydrates—representing 50% and 25% of the total weight, respectively—the most noteworthy ingredients in this regard are flavonoids and methylxanthines [5,6]. Both flavonoids and methylxanthines appear to improve cognitive and cardiovascular function [7,8,9]. The flavonoids that are most often found in cocoa are epicatechin and catechin [10,11]. These flavonoids function as antioxidants through upregulation of nitric oxide production. The methylxanthines that are most often found in cocoa are theobromine and caffeine [10,11]. These act as mild central nervous system stimulants.

Theobromine is the primary methylxanthine found in products made from cacao (Theobroma cacao) [12]. We previously reported that theobromine acts as a phosphodiesterase inhibitor that increases intracellular cyclic adenosine monophosphate (cAMP) [13,14] levels and permeates through the blood–brain barrier [14,15]. Furthermore, cAMP signaling essentially influences the brain mechanism that mediates neural wiring and cognitive processes [16,17,18]. An increase in intracellular cAMP level activates the cAMP-response element-binding protein (CREB), which in turn, leads to the expression of the brain-derived neurotrophic factor (BDNF), one of the neurotrophins that mediates neuronal functions such as learning and memory. We also recently showed that theobromine-fed mice and rats performed better in memory tasks in a CREB/BDNF pathway-dependent manner [14,19].

Chocolate consumption—including dark chocolate—has recently increased around the world [20]. In particular, dark chocolate has become very popular as it contains high concentrations of cacao and may have more beneficial effects on human health compared with normal or milk chocolate [21,22,23]. The cacao content variability (50% to 85%) can generate a one- to seven-fold difference in the epicatechin and catechin content of chocolate, and the epicatechin content has been reported to vary from 4.4 mg per 40 g (50% cacao) to 31.7 mg per 40 g (85% cacao) [24]. Theobromine content also varies, from 75 mg per 50 g in milk chocolate (30% cacao) to 220 mg per 50 g in dark chocolate [10]. In contrast, white chocolate is a cacao-free chocolate and contains few flavonoids and methylxanthine. The taste of white chocolate is not as bitter as that of cacao-based chocolate.

Previous research [7,10] stated that habitual chocolate intake is related to cognitive performance and that frequent chocolate consumption is significantly associated with improved memory. However, little is known about the effects of the habitual dark chocolate intake on cognitive function and neurotrophins in comparison with nondark chocolate intake.

This study aimed to assess the effects of consuming dark chocolate (70% cacao) on cognitive function subchronically in healthy young subjects. We hypothesized that the subchronic consumption of dark chocolate rich in flavonoids and methylxanthines would result in enhanced cognitive function and the release of neurotrophins when compared with the consumption of white chocolate.

## 2. Materials and Methods

### 2.1. Subjects

The experiments were conducted between September and December 2017. The present randomized single-blind study was carried out at Shimane University, Japan. Twenty healthy young subjects (14 men and 6 women, ages 20–31 years old) were recruited from Japanese undergraduate students. Subjects were screened for the following inclusion criteria: nonsmokers, not medicated, and without obvious clinical symptoms and problems, such as hypertension, diabetes, and gastrointestinal disorders. All subjects received no information about their examination data and the exact objective of the study until intervention completion. Also, researchers, including data analysts, were blinded to subject groupings. Subjects were instructed to maintain their habitual diet with the following restrictions: (1) the daily intake of caffeinated beverages—such as coffee, tea, or Japanese tea—had to be ≤3 cups and (2) subjects could not consume any other chocolate except the chocolate that was provided to them. During the experimental period, intense exercise was prohibited. Each subject provided written and informed consent after all procedures and potential risks were explained. All experimental procedures and protocols were approved by the ethics committee of Shimane University (approval number: 2516). Additionally, this study was registered in UMIN (trial registration number: UMIN000029028) on the 6th of September, 2017.

### 2.2. Experimental Protocol

**Figure 1:**
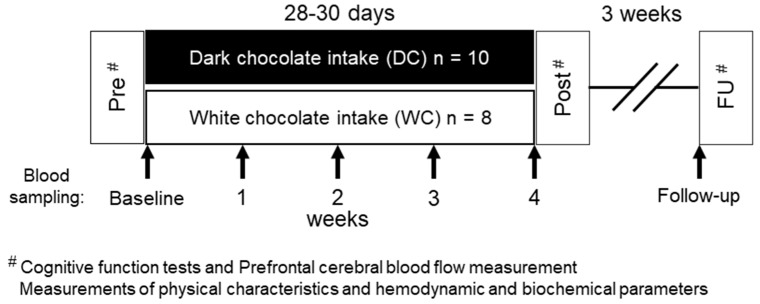
Experiment schedules. Subjects took chocolate daily for 30 days (Chocolate-intake intervention). The subjects’ physical characteristics, cognitive function test, and prefrontal cerebral blood flow (PFCBF) were recorded pre-(Pre) and postintervention (Post) and at a follow-up (FU) visit, 3 weeks after the end of the intervention. Blood was sampled before chocolate intake (baseline), during the chocolate intake intervention (1, 2, 3, and 4 weeks), and 3 weeks after the end of the intervention (FU).

**Figure 2:**
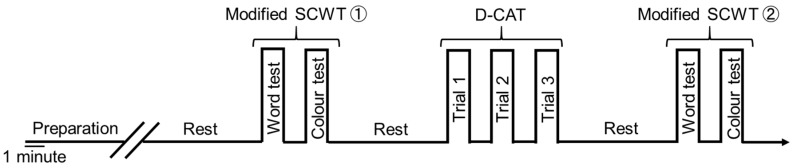
A timeline of cognitive function tests. PFCBF were measured during the cognitive function tests. Preparation, the experimental instruments were attached for subject; rest, 5 min resting time.

The intervention and experiment schedules are summarized in Figure 1. The subjects were randomly divided into two groups using a computer-generated allocation: dark chocolate (DC; *n* = 10) and cacao-free white chocolate (WC; *n* = 10) intake groups. The subjects consumed chocolate daily for 30 days (chocolate-intake intervention). However, two subjects (a man and a woman) in the WC group dropped out of the experiments because of private reasons. Therefore, finally, there were 10 and 8 subjects in the DC and WC groups, respectively. The measurements of physical characteristics, cognitive function tests, and prefrontal cerebral blood flow (PFCBF) were recorded pre-(Pre) and postintervention (Post) and 3 weeks after the end of the intervention (follow-up: FU). FU was evaluated 3 weeks after the end of the intervention because we had preliminarily confirmed that elevated plasma theobromine level from subchronic intake of dark chocolate returned to basal level 3 weeks after the end of the intervention. Between Post and FU, subjects could not intake chocolate, and consuming caffeinated beverages was restricted to the same level as during intervention. Figure 2 shows the details of the cognitive function tests protocol. Hemodynamic and biochemical parameters were analyzed three times, before (baseline) and after chocolate intake intervention, and FU. The plasma theobromine concentrations were measured every week before and during the intervention, and FU. During the experimental period, records of chocolate intake and daily beverages were kept to prevent omitting chocolate intake or drinking too many caffeinated beverages. Adherence to daily chocolate intake was confirmed by the records, which were submitted during weekly visits.

### 2.3. Chocolates and Chocolate-Intake Interventions

The study by Pereira et al. (2019) has shown that dark chocolate, containing 90% cacao, intake for 30 days has beneficial effects on cardiovascular function [25]. However, the taste of dark chocolate is bitter, depending on the content of cacao. In our preliminary trial to take dark chocolate, dark chocolate containing 90% cacao was not able to be taken by our subjects continuously every day. For the experiment, we used dark chocolate containing 70% cacao and cacao-free white chocolate. These chocolates were kindly provided by Dr Inagaki of Morinaga & Co., Ltd, Tokyo, Japan. The components of the chocolate are shown in Table 1. To confirm the theobromine and caffeine amounts, both the dark and white chocolates were analyzed with high-performance liquid chromatography as well as electrospray ionization–mass spectrometry, which was performed with a TSQ quantum mass spectrometer (Thermo Fisher Scientific K.K., Tokyo, Japan) [14,26]. The subjects in each group consumed dark chocolate (24.0 g) or cacao-free white chocolate (24.5 g) every day after lunch for 30 days.

### 2.4. Experimental Conditions for Cognitive Function Tests and PFCBF Measurement

All the subjects visited the laboratory prior to the experiment to become accustomed to the experimental instruments and the cognitive function tests and to reduce learning effects. At Pre, Post, and FU, the subjects visited the laboratory between 12:30 and 16:30, after having a prescribed lunch (200–400 kcal Calorie Mate, Otsuka Pharmaceutical, Japan) and bottle of noncaffeinated barley tea (350 mL). Subjects did not eat chocolate for at least 20 h prior. After urinating, the subjects’ weight and body fat were measured by bioelectrical impedance analysis (DC-320; Tanita, Tokyo, Japan). The subjects then entered an artificial climate chamber that was sound attenuated and controlled to an environmental temperature of 25 °C and a relative humidity of 50%. Subjects rested quietly in a relaxing sitting position for 30 min while the experimental instruments were attached. We measured the systolic blood pressure (SBP), diastolic blood pressure (DBP), and heart rate (HR) using an automatic digital blood pressure monitor (HEM-907; Omron, Kyoto, Japan). Next, the subjects underwent the cognitive function tests (modified SCWT and D-CAT), and their PFCBF responses were measured during the cognitive function tests. The cognitive function tests and PFCBF measurements were independently performed for individuals in the same experimental conditions and were measured by same examiners.

### 2.5. Modified Stroop Color Word Test (Modified SCWT)

The SCWT [27] is a well-known test for examining cognitive performance, for example attention and response inhibition. Therefore, we used a paper version of the SCWT (modified SCWT) that contained Japanese *Kanji* characters for four color words (red, yellow, blue, and green) randomly written in four ink colors (red, yellow, blue, and green) for 10 rows, which comprised a total of 200 characters. The subjects were required to study the paper and read the words (words test) or name the ink colors (colors test) as completely and accurately as possible. The subjects were then instructed to stay awake during the 5 min resting time, after which each subject underwent the (1) word test, and the (2) color test for 1 min each. There was a 1 min resting time between tests (1) and (2). This procedure was repeated in two test trials. Differently arranged color word sheets were used in each trial. We then calculated the average number of correct answers and errors for each set of trials.

### 2.6. Digital Cancellation Test (D-CAT)

The subjects’ prefrontal cortex function was assessed using the D-CAT [28] according to the manual’s protocol. This test uses randomly arranged digit sheets, containing random numbers from 0 to 9 with 50 digits in each row, totaling 600 digits. Subjects were required to search for a given target number(s) and mark them with slashes as completely and accurately as possible within 1 min. This test consisted of three trials: the first trial was a single target number (trial 1), the second required two numbers (trial 2), and the third required three (trial 3). Each trial used differently arranged digit sheets. There were 1 min resting times between the trials. We counted the total number of digits reviewed (total performance) and the total number of digits omitted (total omission). We also calculated the omission ratio (%) using the formula (number of missed targets)/(number of digits inspected) × 100. Total performance index pertains mainly to cognitive components information processing speed, focused attention, and sustained attention, and omission ratio primarily reflects sustained and selective attention [28].

### 2.7. Prefrontal Cerebral Blood Flow (PFCBF)

During the cognitive function tests, we measured the PFCBF using near-infrared spectroscopy, which has a two-channel wireless system (Pocket NIRS; DynaSense Inc., Hamamatsu, Japan). Concentration changes of oxygenated hemoglobin (Oxy-Hb) were measured by near-infrared light emitted from laser photodiodes with three different wavelengths (735, 810, and 850 nm), which penetrate brain tissue. The distance between the emission and the corresponding receiving sensor was 3 cm. Therefore, we measured at a point 3 cm beneath the scalp, which is the surface of the cerebral cortex. Two probes (PProbe-1M; DynaSense Inc. Hamamatsu, Japan); were attached to the frontal surface of the head at the Fp1 and Fp2 positions, following the 10/20 international system of electrode placement. These probes were covered with a headgear, to which soft black sponges to shield the probes from light were attached. Baseline data were collected after the 5 min resting time before the tests, starting with the subjects looking at a cross mark. PFCBF data were stored in a computer (model dynabook Qosimio G50/98; Toshiba Co., Tokyo, Japan) at 500 Hz using a data acquisition system (UAS-308S; Unique medical Co., Tokyo, Japan). The data were acquired at a sampling rate of 10 Hz and were first sent to a PC (model CF-SZ6; Panasonic, Osaka, Japan) through a wireless system (Bluetooth^TM^, Kirkland, WA, USA) and then digitized through a module (USB-3100; Measurement Computing Co., Norton, MA, USA). We calculated the data from the average of two probe channels—left side and right side—every 5 s. The results show the change in the baseline data from the average value for 1 min at rest (adjacent 1 min before test started).

### 2.8. Blood Samples

On the day before the blood sampling, the subjects were instructed to have a prescribed food (500 kcal of commercially available balanced food) as a dinner and not to eat anything after 20:00. Additionally, the subjects were prohibited from consumption of alcoholic beverages and high-intensity exercise. On the day of the blood sampling, the subjects were not allowed to have a breakfast and instructed to visit the laboratory between 8:00 and 10:00 after drinking a bottle of water (280 mL). After urinating, the subjects entered a laboratory room that was controlled at an ambient temperature of 25 °C and a relative humidity of 50%. After resting for at least 20 min, blood samples were taken from the vein at the cubital region at a volume of 10 mL for all biochemical analysis or 3 mL for theobromine assay only.

### 2.9. Biochemical Analyses

Part of the blood analyses was outsourced (Japan Clinical Laboratories Inc., Kyoto, Japan). Plasma osmolality (Osm) was determined by freezing point depression. The sodium, potassium, and chlorine levels of plasma were measured by an ion-selective electrode method. The TP and Alb were measured by the biuret and bromocresol green methods, respectively. Glucose, triglyceride (TG), and free fatty acid (FFA) levels were measured by using an enzymatic method.

### 2.10. Measurements of Theobromine and Caffeine Content in Plasma

We mixed 50 μL of plasma with 250 ng of caffeine-d9 in 200 μL of acetonitrile and then kept the samples at −30 °C for 30 min. The samples were centrifuged at 5000× *g* for 10 min at 4 °C to remove the precipitated proteins. The supernatants were analyzed with high-performance liquid chromatography in combination with electrospray ionization–mass spectrometry that was performed with a TSQ quantum mass spectrometer (Thermo Fisher Scientific K.K., Tokyo, Japan). The high-performance liquid chromatography was performed with a Luna 3 μm C18(2) 100 Å liquid chromatography column (100 × 2.0 mm, Phenomenex, Inc., Torrance, CA, USA) at 30 °C. The samples were eluted in a mobile phase consisting of acetonitrile–methanol (4:1, *v*/*v*) and water–acetic acid (100:0.1, *v*/*v*) in a 10:90 ratio for 2 min. After 5 min, the ratio was changed to 70:30 and maintained for 7 min. Subsequently, the ratio was changed to 80:20 and held for 2 min. Finally, after 9 min, the ratio was changed to 100:0 and held for 2 min with a flow rate of 0.1 mL/min. Tandem mass spectrometry analyses were conducted in the positive ion mode, and theobromine (*m*/*z* 180.8N163.1), caffeine (*m*/*z* 195.1N138.1), and caffeine-d9 (*m*/*z* 204.2N144) were detected and quantified with selected reaction monitoring. The peaks were selected, and their areas were calculated with Xcalibur™ 2.1 software (Thermo Fisher Scientific K.K.) [14,29].

### 2.11. Enzyme-Linked Immunosorbent Assay (ELISA)

Plasma NGF and BDNF levels were evaluated by using ELISA. Equal amounts of plasma were analyzed with the NGF Rapid ELISA kit: Human (BEK-2212-1P; Biosensis Pty Ltd., Thebarton, SA, Australia) and BDNF Rapid ELISA kit (BEK-2211-1P; Biosensis Pty Ltd.) according to the manufacturer’s protocol. Absorbance at 450 nm was measured by using a plate reader (DTX880, Beckman Coulter, CA, USA) and each neurotrophin concentration was calculated using SoftMax pro software (Molecular Devices, LLC, San Jose, CA, USA) as described previously [14].

### 2.12. Outcome Measures

In this study, the primary outcomes were improvement of cognitive function by DC ingestion, estimated using the paper version of the SCWT (modified SCWT) and the D-CAT. Secondary outcome was elevated neurotrophin concentration in the plasma.

### 2.13. Statistics

Analyses were performed using IBM SPSS Statistics v23 (Armonk, NY, USA) for windows. Unpaired *t* test was applied for examining any significant differences in the physical characteristics and blood chemical measures between the groups at Pre (Supplemental Tables S1 and S2). Paired *t* test was used to examine any significant differences in the physical characteristics and blood chemical measures within group; Pre vs. Post and Pre vs. FU (Supplemental Tables S1 and S2). This model was also used to examine any significant differences in cognitive function test results, neurotrophin concentration; Pre vs. Post and Pre vs. FU. A repeated measurement analysis of variance (ANOVA) was used to examine any significant differences in theobromine concentration in the plasma. As a subsequent post hoc test, the Tukey–Kramer test was used to perform any pairwise comparisons. Two-way ANOVA (intervention× time) for repeated measures was used to examine any significant differences in the variables during the cognitive function tests; Pre vs. Post and Pre vs. FU in each group. The null hypothesis was rejected when *p* < 0.05.

## 3. Results

### 3.1. Baseline Scores

There were no statically significant baseline (Pre) differences between groups for any measurements, i.e., physical characteristics, neurotrophin concentrations, hemodynamic parameters, and cognitive function.

### 3.2. Adherence to Daily Chocolate Consumption and Intake of Caffeinated Beverages

Adherence to the daily chocolate intake during the intervention period was 99.4 ± 0.4% and 97.5 ± 1.1% in the DC and WC groups, respectively. These results were reliable enough to assess the effects of chocolate consumption on cognitive function. The total amount of intake of caffeinated beverages during the intervention was 26 cups (0.8 ± 0.2 cups/day) and 38 cups (1.2 ± 0.3 cups/day) in the DC and WC groups, respectively. There were no significant differences between groups (*F* (1, 16) = 2.66, *p* = 0.27).

### 3.3. Physical Characteristics and Appearances, Hemodynamic Parameters, and Biochemical Analysis

Supplementary Tables S1 and S2 show the physical characteristics, hemodynamic parameters, and biochemical analysis at Pre, Post, and Fu in each DC and WC group. The DC intake group showed a slight increase in weight, body fat, and body mass index at FU, but not at Pre and Post, whereas the WC intake group showed no change in these parameters at Pre, Post, or FU. Neither DC nor WC intake altered the hemodynamic parameters at Pre, Post, or FU.

DC intake slightly decreased chlorine and calcium concentrations at Post and FU. In the WC group, γ-GTP, total protein (TP), and albumin concentrations (Alb) were slightly decreased at Post and FU. Calcium concentration was slightly decreased at FU in the WC group.

There were several differences between the two groups concerning physical characteristics and biochemical parameters (Supplementary Tables S1 and S2). However, those differences were nonsignificant and all data and parameters remained within normal range. Therefore, it can be said that the intervention did not induce dramatic changes in physical characteristics or hemodynamic and biochemical parameters during the experimental period, including FU.

### 3.4. Cognitive Function Tests

**Figure 3:**
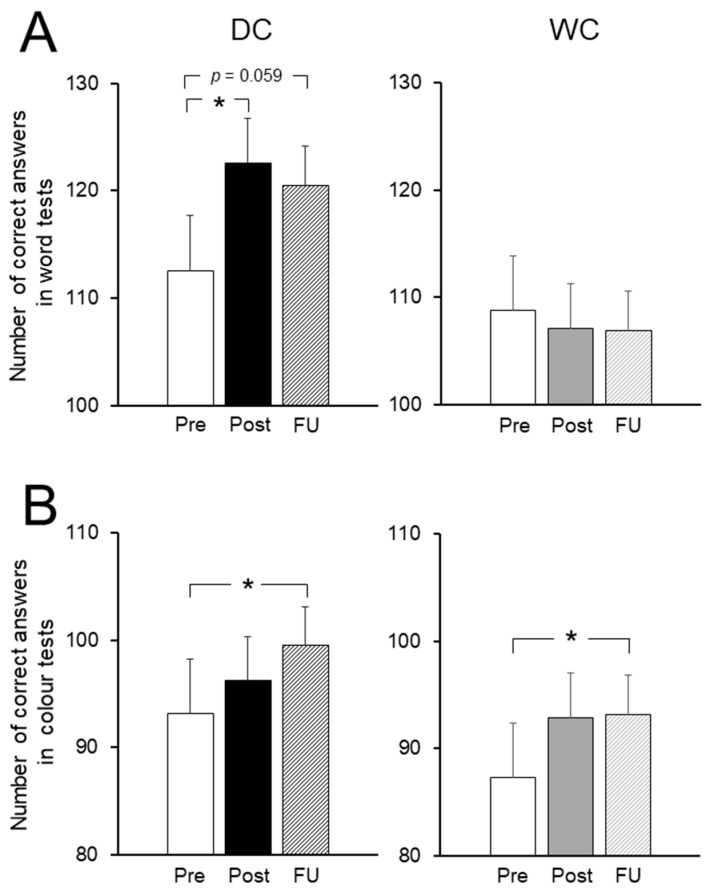
The effects of dark chocolate (DC) and white chocolate (WC) intake on performance in the modified SCWT. The number of correct answers in word tests in the modified SCWT at Pre, Post, and FU for the DC group ((**A**), left panel) and the WC group ((**A**), right panel). The number of correct answers in the color tests at Pre, Post, and FU for the DC group ((**B**), left panel) and the WC group ((**B**), right panel). Values are the means ± SEM. * Significant differences vs. Pre at *p <* 0.05.

We investigated the effect of dark chocolate intake with both word tests (Figure 3A) and color tests (Figure 3B) by using a modified Stroop color word test (SCWT) during the experiment period and FU. DC intake significantly increased the number of correct answers at Post compared with those at Pre (*t* (9) = 2.93, *p* = 0.017). At FU, the number of correct answers from the DC group remained at a higher level than those at Pre, but not significantly so (*t* (9) = 2.16*, p* = 0.059) (Figure 3A, left panel). WC intake did not affect the number of correct answers during the experimental period, including FU (Pre vs. Post, *t* (7) = 0.27*, p* = 0.80; Pre vs. FU, *t* (7) = 0.32*, p* = 0.76) (Figure 3A, right panel). However, both the DC and WC groups showed a significant increase in the number of correct answers in the color tests at FU compared with those at Pre (DC, *t* (9) = 3.11, *p* = 0.012; WC, *t* (7) = 2.37, *p* = 0.050) (Figure 3B). For both the DC and WC intake groups, there were no changes in the number of erroneous answers during the experimental period (*p* > 0.14, all) (Supplementary Table S3). Overall, the DC group was found to perform better than the WC group in the modified SCWT.

Next, we investigated the effect of DC intake on total performance and omission ratio by using a digital cancellation test (D-CAT) (Table 2). DC intake significantly increased the number of total performance in trial 3 at Post compared with those at Pre (*t* (9) = 3.04, *p* = 0.014). Also, at FU, the total performance from the DC group remained at a higher level than those at Pre (*t* (9) = 2.67*, p* = 0.026) (Table 2 upper). WC intake did not affect the number for total performance in trial 3 during the experimental period, including FU (Pre vs. Post, *t* (7) = 1.48*, p* = 0.18; Pre vs. FU, *t* (7) = 0.75*, p* = 0.48) (Table 2 lower). Neither DC nor WC intake altered the omission ratio in trial 3 during the experimental period (*p* > 0.11). Similar results were not observed in total performance in trial 1 and 2 of the DC intake group (*p* > 0.20, for all). However, omission ratio in the DC group were significantly decreased at Post in both trial 1 (*t* (9) = 2.79, *p* = 0.021) and trial 2 (*t* (9) = 2.51, *p* = 0.033) compared with those at Pre.

### 3.5. Prefrontal Cerebral Blood Flow (PFCBF)

**Figure 4:**
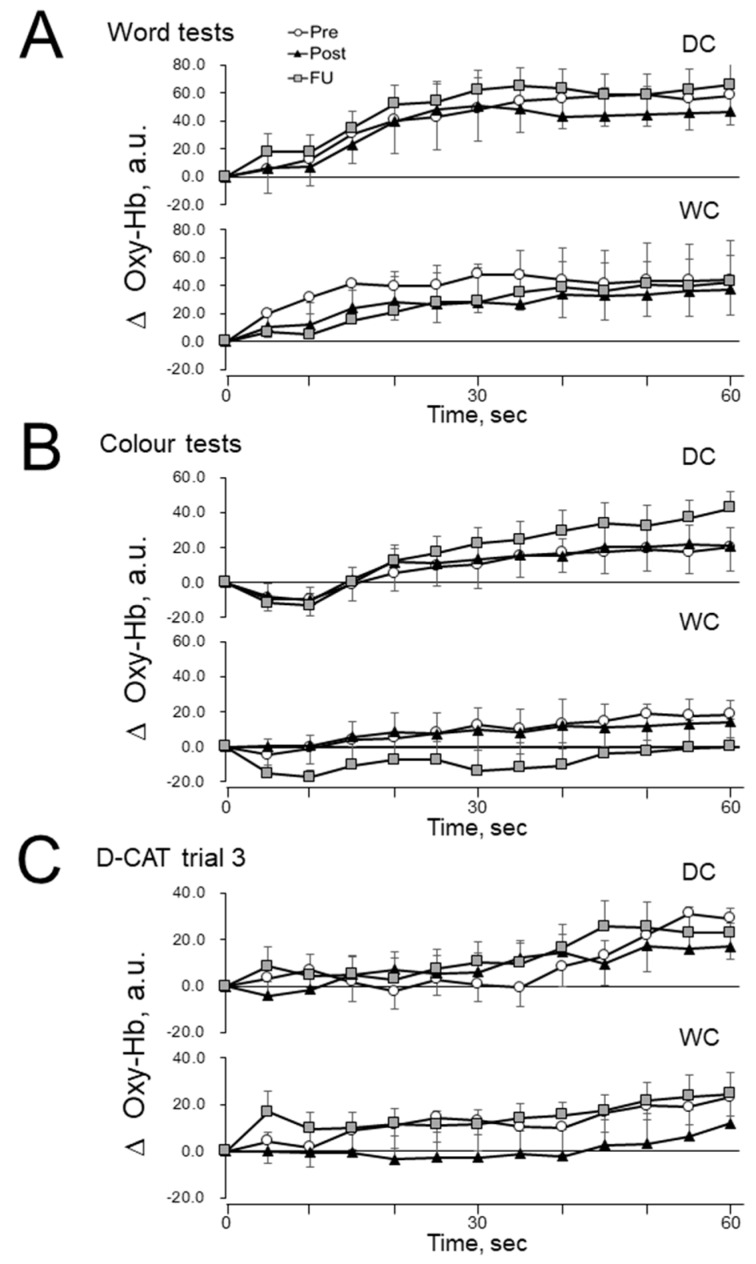
The effects of dark chocolate (DC) and white chocolate (WC) intake on PFCBF during the cognitive function tests. Average of oxygenated hemoglobin (Oxy-Hb) during the modified SCWT in word tests at Pre, Post, and FU for the DC group ((**A**), upper panel) and WC group ((**A**), lower panel). Average of Oxy-Hb during the modified SCWT in color tests at Pre, Post, and FU for the DC group ((**B**), upper panel) and the WC group ((**B**), lower panel). Average of Oxy-Hb during the D-CAT at Pre, Post, and FU for the DC group ((**C**), upper panel) and the WC group ((**C**), lower panel). Variables are expressed as changes (∆) from the baseline at the rest period in each trial. Means ± SEM bars are presented every 5 s.

Figure 4 shows the concentration change in oxygenated hemoglobin (Oxy-Hb) in the prefrontal cortex. This is an indicator of changes in regional cerebral blood flow during the modified SCWT (Figure 4A,B) and the D-CAT in trial 3 (Figure 4C) at Pre, Post, and FU for both the DC and WC groups. There were no significant differences in PFCBF among Pre, Post, and FU in either group (*p* > 0.05 for all). Variables are expressed every 5 s as changes (∆) from the values at the rest period in each trial.

### 3.6. Plasma Nerve Growth Factor (NGF) and Brain-Derived Neurotrophic Factor (BDNF) Levels

**Figure 5:**
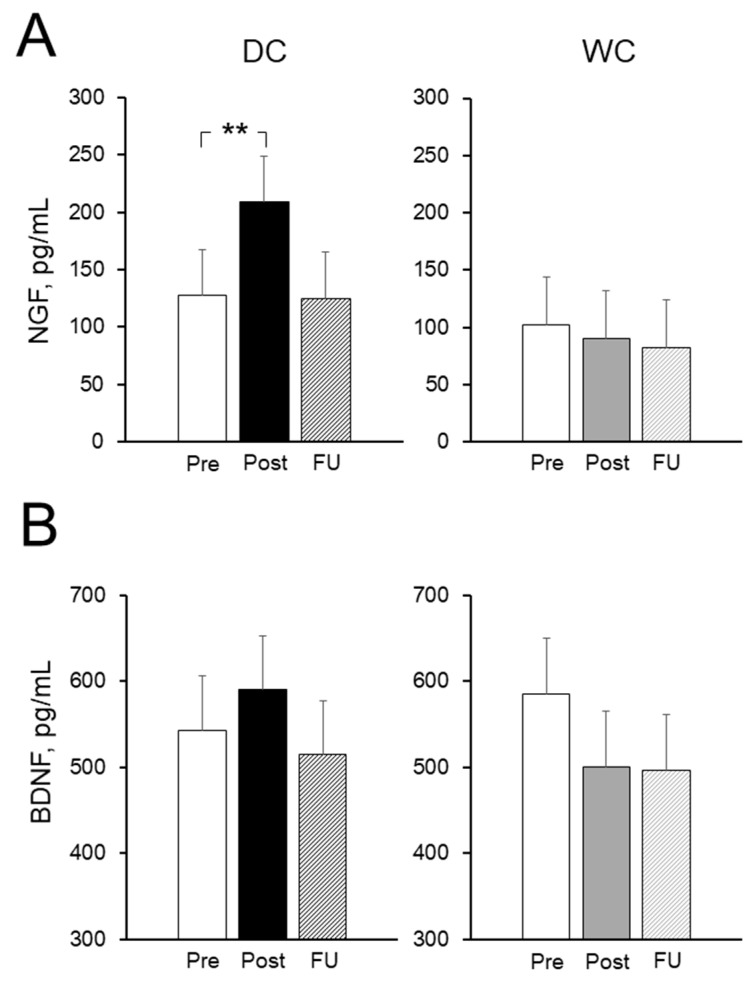
The effects of dark chocolate (DC) and white chocolate (WC) intake on neurotrophins in the plasma. The NGF concentrations in plasma at Pre, Post, and FU for the DC group ((**A**), left panel) and the WC group ((**A**), right panel). The BDNF concentrations in plasma at Pre, Post, and FU for the DC group ((**B**), left panel) and the WC group ((**B**), right panel). Values are the means ± SEM. ** Significant differences vs. Pre at *p <* 0.01.

Figure 5 shows the changes in the concentrations of NGF and BDNF at Pre, Post, and FU for both the DC and WC groups. For the DC group, the level of NGF was increased at Post (*t* (9) = 3.59, *p* = 0.0059) and returned to baseline at FU (*t* (9) = 0.35*, p* = 0.74) (Figure 5A). In the WC intake group, the level of NGF did not change throughout the intervention period, including FU (Pre vs. Post, *t* (7) = 1.46*, p* = 0.19; Pre vs. FU, *t* (7) = 1.16*, p* = 0.28). This means that neither DC nor WC intake altered BDNF concentration levels during the experimental period, including FU (*p* > 0.17 for all) (Figure 5B).

### 3.7. Theobromine and Caffeine Concentrations in Plasma

**Figure 6:**
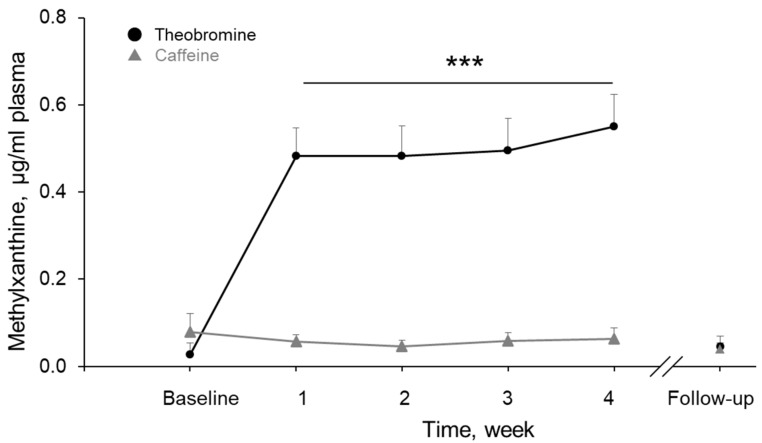
Changes in theobromine and caffeine concentrations in plasma of the DC group during the experimental period, including follow-up. Values are the means ± SEM. *** Significant differences vs. baseline at *p <* 0.001.

Figure 6 shows the changes of plasma theobromine and caffeine concentrations in the DC group. The theobromine concentration in plasma increased significantly and remained high during the DC intervention period (*t* (9) > 6.71, *p* < 0.001, for all). It then returned to the baseline level at follow-up (*t* (9) = 0.80, *p* = 0.44). In the WC group, no theobromine was detected in plasma through the intervention period. The caffeine concentration in plasma did not change through the study, including at FU (*t* (9) < 1.41, *p* > 0.20, for all).

## 4. Discussion

Several existing studies [1,2,7,8,9,10,11,30,31,32] have shown that chocolate intake may have beneficial effects on health, including cardiovascular function, cholesterol metabolism, and cognitive function. Notably, habitual chocolate intake has been linked to cognitive performance [10]. However, little has been known about the effects of the subchronic consumption of dark chocolate on cognitive function and plasma neurotrophins. Our results show that dark chocolate intake (Post) increases NGF and theobromine levels in plasma and enhances cognitive function and performance. However, three weeks after the end of chocolate intake (FU), NGF and theobromine levels in plasma were returned to the Pre levels. Interestingly, the dark chocolate-mediated enhancement of cognitive function was observed three weeks after the end of chocolate intake. Thus, there is a possibility that NGF promotes neuronal plasticity via subchronic consumption of dark chocolate and improves cognitive performance. This observation disappeared three weeks after the end of chocolate intake, but the effect of improves cognitive performance at Post persisted in FU because neuronal plasticity, such as neurogenesis or/and synaptogenesis, might be performed in Post.

In contrast, white chocolate consumption did not affect NGF and theobromine levels in plasma or cognitive function. These results suggested that subchronic consumption of dark chocolate has beneficial effects on health by enhancing cognitive function.

To evaluate the beneficial effects of subchronic consumption of dark chocolate on cognitive function, a variety of cognitive and behavioral processes—including elective attention, inhibition, and interference control—were assessed by modified Stroop color word test (SCWT) and a digit cancellation task (D-CAT). The results showed that the subchronic consumption of dark chocolate significantly enhanced performance in the modified SCWT and the D-CAT, indicating the beneficial effects of dark chocolate on cognition. Interestingly, the dark chocolate-mediated enhancement of cognitive function was observed in the modified SCWT and D-CAT three weeks after the end of chocolate intake. This indicates that the subchronic effect of dark chocolate on cognitive function lasts several weeks after the consumption of dark chocolate has ceased. We have previously confirmed that theobromine-mediated enhancement of motor learning persisted for at least 30 days after ceasing oral theobromine intake in mice [14].

The significant ingredients of dark chocolate in this case are flavonoids (e.g., epicatechin) and methylxanthines (e.g., theobromine). The concentrations of epicatechin and theobromine in human blood plasma peak at approximately 0.5–3 h post consumption [10,33]. Both epicatechin and theobromine have been shown to cross the blood–brain barrier in animal studies [14,15,34], suggesting that the flavonoids and methylxanthines from chocolate have the ability to act directly on the brain, which could potentially lead to cognitive enhancement. In their work, both Massee et al. [30] and Smit et al. [31] showed that the acute effects of high doses of cocoa flavonoids and methylxanthines in dark chocolate improved cognitive performance in humans. Unfortunately, Massee et al. [30] could not show the subchronic effects of high doses of cocoa flavonoids on cognitive enhancement. However, this study has evaluated and confirmed the subchronic beneficial effects of dark chocolate on cognitive function. Following the above, the more significant ingredients of dark chocolate affecting subchronical human cognitive health are methylxanthines, especially theobromine, rather than flavonoids, since it has been previously confirmed that theobromine-mediated cognitive enhancements persisted for several weeks in mice [14].

In this study, we measured the concentration of theobromine—but not epicatechin—in plasma. During the intervention (dark chocolate intake), the concentration of theobromine in plasma increased gradually and reached a level of ca. 0.5 μg/mL, where it held steady. After ceasing the DC intake, it returned to the baseline level. Conversely, white chocolate did not increase the concentration of theobromine in plasma. These results indicate that dark chocolate was satisfactorily absorbed, exerted its beneficial effects, and was then excreted in our subjects.

Neurotrophins—including NGF and BDNF—are known to play critical roles in neuroprotective and growth-promoting effects [35,36,37]. Both NGF and BDNF bind with tropomyosin receptor kinase (Trk) receptors—TrkA and TrkB—and activate downstream signaling cascades [36,37] to improve cognitive function [38,39]. In the present study, the subchronic consumption of dark chocolate showed an increase in NGF levels in plasma, but there was no change in BDNF levels (Figure 5). We cannot confirm that plasma NGF is responsible for dark chocolate-mediated cognitive enhancements, since NGF is not able to freely cross the blood–brain barrier. However, neither can we confirm that dark chocolate-mediated cognitive enhancements are unrelated to NGF and BDNF levels in the human brain, since we are not able to determine the levels of NGF and BDNF in the human brain following dark chocolate intake. However, theobromine—which dark chocolate is rich in—was found to increase the level of BDNF in mouse and rat brains [14,19], implying that dark chocolate may upregulate NGF and/or BDNF in the human brain and improve cognitive function.

Dark chocolate may not only exert direct effects on the brain but could potentially act indirectly through blood supply. In previous studies, an increase in cerebral blood flow was observed following cocoa supplementation [32,40,41,42,43], suggesting that it is possible for cocoa to improve cognitive performance indirectly through improvements to cerebral blood flow. However, we did not observe an increase in blood supply in the forebrain during the cognitive tasks following DC intake in this study (Figure 4). Furthermore, the blood pressure and heart rate of subjects were not altered by the DC consumption (Supplementary Table S1).

We need to mention herein the fact that there are some limitations that deserve additional discussion. Firstly, this study includes a relatively small sample size. Given the properties of a randomized human interventional trial, a small sample size may induce some bias and great variance in the effects. For example, plasma BDNF level and PFCBF, which were insignificantly different between DC and WC group, could be due to a relatively small sample size. Secondly, the controlled diet in this experiment was only the dinner one day before the sampling blood and the lunch just before the cognitive function tests. For other days during the intervention, we gave instructions to the subjects that maintain their habitual diet during the experiment period, and did not have detailed data for subjects’ meals during the experiment. Lack of dietary survey during the experimental period may have caused some bias in the effect of dark chocolate on the cognitive performance or other biological measurements. Thirdly, in the modified SCWT (color test), both the DC and WC groups increased in the number of correct answers at FU compared with Pre. This result may be induced by the learning effect of performing after repetition of the same test. This effect, however, might also have indicated a potential bias such as a difference in learning ability of individuals and/or the effects of daily diet, care should be taken in the interpretation of results. Fourth, we did not investigate exact physical activity, e.g., metabolic equivalents, during intervention, and further research will need to take this into account given the influence of the physical activity on cognitive function [44,45]. Fifth, since this work has been planned as a single-blind study, well-designed research such as a double-blind trial is required in a future. Nonetheless, we believe that dark chocolate contributed toward improving cognitive performance even in young subjects and our results at least partly support previous reports that show a beneficial effect of dark chocolate on human cognitive function [25,30,31]. Future studies that seek to investigate should objectively evaluate the effects of dark chocolate intake with accurate diet and physical activity control and a larger number of subjects.

In conclusion, the subchronic consumption of dark chocolate was found to enhance cognitive performance and increase NGF in plasma. Future research should investigate the molecular mechanism of cognitive enhancements related to dark chocolate.

## Figures and Tables

**Table 1:** Nutritional components of dark chocolate (DC) and white chocolate (WC).

	DC (24.0 g/Day)	WC (24.5 g/Day)
Energy, kcal	135	145
Protein, g	2.0	2.0
Fat, g	10.0	9.5
Carbohydrates, g	9.0	12.5
Cacao polyphenol, mg	540.0	ND
Epicatechin, mg	34.8	ND
Caffeine, mg	26.8	ND
Theobromine, mg	197.5	ND

ND: not detected.

**Table 2:** D-CAT at Pre, Post and FU for the DC and WC groups.

	**DC (n = 10)**								
	**Trial 1**			**Trial 2**			**Trial 3**		
	**Pre**	**Post**	**FU**	**Pre**	**Post**	**FU**	**Pre**	**Post**	**FU**
TP, count	380 ± 18	359 ± 18	372 ± 18	288 ± 16	275 ± 14	286 ± 13	218 ± 15	233 ± 13 *	230 ± 12 *
Omission ratio, %	5.1 ± 1.4	1.6 ± 1.0 *	3.1 ± 1.5	8.1 ± 1.9	4.0 ± 1.6 *	6.2 ± 2.1	7.3 ± 1.9	5.8 ± 1.7	4.9 ± 1.5
	**WC (n = 8)**								
	**Trial 1**			**Trial 2**			**Trial 3**		
	**Pre**	**Post**	**FU**	**Pre**	**Post**	**FU**	**Pre**	**Post**	**FU**
TP, count	329 ± 10	349 ± 10	335 ± 21	264 ± 12	272 ± 14	264 ± 20	211 ± 14	221 ± 15	217 ± 20
Omission ratio, %	1.5 ± 1.2	3.1 ± 1.1	1.8 ± 1.1	4.0 ± 1.1	1.9 ± 0.5	2.2 ± 0.8	5.5 ± 2.1	5.1 ± 1.3	4.4 ± 1.7

TP: total performance; values are the means ± SEM. * Significant differences vs. Pre in each trial at *p <* 0.05.

## Supplementary Materials

The following are available online at https://www.mdpi.com/2072-6643/11/11/2800/s1, Table S1: Physical characteristics of subjects at Pre, Post, and FU; Table S2: Biochemical analysis at Pre, Post, and FU; and Table S3: Number of error answers in word and color tests of modified SCWT for the DC and WC group.
