# Supplementary material for: Sub-Chronic Consumption of Dark Chocolate Enhances Cognitive Function and Releases Nerve Growth Factors: A Parallel-Group Randomized Trial
Source: Nutrients. 2019 Nov 16;11(11):2800. doi: 10.3390/nu11112800 (PMC6893800; doi:10.3390/nu11112800)
Supplement: Supplementary file 1 [file nutrients-11-02800-s001.docx]

***Supplemental Table 1.*** *Physical characteristics Pre and Post chocolate intake and Follow-up (FU).*

|  | DC (n=10) | | |  | WC (n=8) | | |
| --- | --- | --- | --- | --- | --- | --- | --- |
|  | Pre | Post | FU |  | Pre | Post | FU |
| Height, cm | 168.6 ± 1.9 | － | － |  | 169.2 ± 2.2 | － | － |
| Weight, kg | 61.3 ± 3.6 | 61.9 ± 3.7 | 62.2 ± 3.7* |  | 60.1 ± 2.7 | 60.6 ± 2.6 | 60.5 ± 2.8 |
| Body fat, % | 20.7 ± 1.5 | 21.0 ± 1.5 | 21.4 ± 1.5* |  | 18.5 ± 1.9 | 18.6 ± 2.2 | 18.1 ± 2.4 |
| BMI, kg/m^2^ | 21.4 ± 0.9 | 21.6 ± 0.9 | 21.7 ± 0.9* |  | 21.0 ± 0.8 | 21.1 ± 0.8 | 21.1 ± 0.8 |
| SBP, mmHg | 122 ± 3 | 123 ± 3 | 124 ± 3 |  | 122 ± 3 | 124 ± 3 | 122 ± 4 |
| DBP, mmHg | 68 ± 2 | 68 ± 2 | 69 ± 3 |  | 66 ± 2 | 69 ± 2 | 67 ± 3 |
| HR, bpm | 69 ± 3 | 71 ± 3 | 70 ± 5 |  | 69 ± 6 | 70 ± 4 | 68 ± 5 |

Values are expressed as means ± SE. BMI, body mass index; SBP and DBP, systolic, and diastolic blood pressures, respectively; HR, heart rate. *Significant differences vs. Pre at *P<*0.05.

|  | DC (n=10) | | |  | WC (n=8) | | |
| --- | --- | --- | --- | --- | --- | --- | --- |
|  | Pre | Post | FU |  | Pre | Post | FU |
| Ht, % | 45.6 ± 1.4 | 45.7 ± 1.4 | 45.3 ± 1.5 |  | 45.6 ± 1.2 | 45.0 ± 1.2 | 44.5 ± 1.1 |
| AST, U/L | 21.5 ± 3.4 | 21.4 ± 1.1 | 23.2 ± 2.6 |  | 19.1 ± 1.6 | 17.6 ± 1.3 | 18.8 ± 0.8 |
| ALT, U/L | 14.7 ± 1.7 | 16.7 ± 3.1 | 17.3 ± 2.8 |  | 15.1 ± 2.1 | 14.8 ± 2.1 | 13.9 ± 1.6 |
| ALP, U/L | 189.3 ± 15.6 | 189.9 ± 17.4 | 190.8 ± 15.8 |  | 217.8 ± 13.2 | 240.3 ± 24.9 | 223.1 ± 14.3 |
| γ-GTP, U/L | 17.1 ± 1.5 | 18.8 ± 2.2 | 18.5 ± 2.1 |  | 18.0 ± 1.3 | 16.5 ± 1.2* | 16.5 ± 1.1 |
| TP, g/dL | 7.31 ± 0.1 | 7.1 ± 0.1 | 7.2 ± 0.1 |  | 7.7 ± 0.1 | 7.4 ± 0.1** | 7.4 ± 0.1* |
| Alb, g/dL | 4.7 ± 0.1 | 4.6 ± 0.1 | 4.6 ± 0.0 |  | 4.8 ± 0.1 | 4.6 ± 0.1*** | 4.6 ± 0.1* |
| TG, mg/dL | 79.6 ± 13.8 | 73.0 ± 10.3 | 72.3 ± 11.3 |  | 95.1 ± 24.4 | 96.9 ± 25.6 | 88.3 ± 16.1 |
| TC, mg/dL | 172.8 ± 9.6 | 167.4 ± 5.7 | 172.8 ± 4.2 |  | 175.4 ± 14.9 | 174.8 ± 13.9 | 169.0 ± 13.3 |
| FFA, µEq/L | 556.8 ± 38.1 | 587.2 ± 66.1 | 568.2 ± 78.2 |  | 510.4 ± 68.9 | 441.8 ± 78.6 | 445.9 ± 64.9 |
| Na, mEq/L | 141.1 ± 0.2 | 140.5 ± 0.4 | 139.8 ± 0.5 |  | 141.1 ± 0.6 | 141.0 ± 0.4 | 141.6 ± 0.3 |
| Cl, mEq/L | 104.0 ± 0.5 | 101.5 ± 0.5** | 101.7 ± 0.5** |  | 102.6 ± 0.4 | 101.8 ± 0.6 | 102.9 ± 0.5 |
| K, mEq/L | 4.0 ± 0.1 | 4.2 ± 0.1 | 4.2 ± 0.0* |  | 4.1 ± 0.1 | 4.3 ± 0.1 | 4.2 ± 0.2 |
| Ca, mg/dL | 9.4 ± 0.0 | 9.1 ± 0.1** | 9.0 ± 0.1*** |  | 9.6 ± 0.1 | 9.2 ± 0.1 | 9.0 ± 0.1** |
| BG, mg/dL | 87.3 ± 1.3 | 90.0 ± 1.5 | 90.4 ± 1.9 |  | 88.4 ± 2.3 | 91.3 ± 1.5 | 92.5 ± 2.4 |
| Osm, mOsm/L | 279.7 ± 1.0 | 280.6 ± 0.8 | 280.1 ± 0.8 |  | 279.8 ± 1.0 | 281.8 ± 0.8 | 281.8 ± 1.3 |
| COR, µg/dL | 11.4 ± 0.7 | 12.3 ± 0.6 | 12.3 ± 1.0 |  | 11.8 ± 1.2 | 11.5 ± 0.9 | 12.4 ± 0.9 |

***Supplemental Table 2.*** *Biochemical analysis Pre and Post chocolate intake and Follow-up (FU).*

Values are expressed as means ± SE. Ht, hematocrit; AST, aspartate aminotransferase; ALT, alanine aminotransferase; ALP, alkaline phosphatase; γ-GTP, γ-glutamyl transpeptidase; TP, total protein; Alb, albumin; TG, triglycerides; TC, total cholesterol; FFA, free fatty acid; Na, sodium; Cl, chlorine; K, potassium; Ca, calcium; BG, blood glucose; Osm, osmotic pressure; COR, cortisol.

*, **, ***, Significant differences vs. Pre in each group at *P<*0.05, *P<*0.01, *P<*0.001.

***Supplemental Table 3.*** *Number of error answers in word and colour tests of modified SCWT for the DC and WC group.*

|  | DC (n=10) | | |  | WC (n=8) | | |
| --- | --- | --- | --- | --- | --- | --- | --- |
|  | Pre | Post | FU |  | Pre | Post | FU |
| Word tests, count | 0.6 ± 0.1 | 0.4 ± 0.2 | 1.1 ± 0.5 |  | 0.6 ± 0.2 | 0.9 ± 0.6 | 0.9 ± 0.5 |
| Colour tests, count | 0.8 ± 0.2 | 0.9 ± 0.4 | 0.9 ± 0.2 |  | 0.8 ± 0.3 | 0.3 ± 0.2 | 0.5 ± 0.2 |

Values are expressed as means ± SE.
